# Tapering Levothyroxine Dose for Intra-Amniotic Infusion in the Antenatal Treatment of Fetal Goiter: A Case Report

**DOI:** 10.1155/crog/9819796

**Published:** 2025-10-30

**Authors:** Wendy Lin, Omar Abuzeid

**Affiliations:** ^1^Department of Medicine, Chicago College of Osteopathic Medicine, Midwestern University, Downer's Grove, Illinois, USA; ^2^Department of Maternal Fetal Medicine, Franciscan Health Crown Point, Crown Point, Indiana, USA

**Keywords:** case report, fetal goiter, fetal hypothyroidism, intra-amniotic infusion, levothyroxine

## Abstract

Fetal goitrous hypothyroidism is associated with important obstetrical complications including preterm birth, polyhydramnios, respiratory disorders, and neurodevelopmental impairments. There are currently no standard treatment guidelines for fetal goitrous hypothyroidism, and further studies are needed to help establish treatment guidelines. We report a case of a healthy 41-year-old female whose fetus was diagnosed with fetal goiter at 20 weeks gestation. The patient underwent weekly intra-amniotic infusions of levothyroxine, and the fetal goiter resolved by 30 weeks gestation. The infant was delivered vaginally at 36 weeks with no evidence of goiter on physical exam and diagnosed with congenital hypothyroidism upon follow-up with pediatric endocrinology. Both mother and infant are doing well today with the infant showing no signs of neurodevelopmental impairment. This case demonstrates that intra-amniotic infusion of levothyroxine for fetal goiter may improve perinatal outcomes.

## 1. Introduction

Fetal goiter is characterized by enlarged fetal thyroid gland size due to defects in thyroid hormone synthesis or transportation [1]. It can occur in hypothyroid, hyperthyroid, or euthyroid states [2]. In the case of hypothyroid states, it is termed fetal goitrous hypothyroidism [2]. Causes of fetal goiter include genetic abnormalities, maternal thyroid conditions, exposure to maternal antithyroid medications, and maternal iodine deficiencies [3]. Fetal goitrous hypothyroidism is associated with important obstetrical complications including preterm birth, polyhydramnios, respiratory disorders, and neurodevelopmental impairments [4]. A rare condition, fetal goitrous hypothyroidism, affects between 1:30,000 and 1:50,000 newborns in North America and Europe [5]. It is also important to consider differential diagnoses of fetal goiter, including fetal hyperthyroidism, teratoma, cystic hygroma/lymphangioma, hemangioma, and epignathus [6]. There is currently no standard treatment method for fetal goitrous hypothyroidism, and prenatal treatment remains controversial [6]. While there have been a few reported cases that show successful reduction of fetal goiter with intra-amniotic infusions of levothyroxine, more literature is needed to help establish guidelines for standard treatment [3]. Herein, we report a case of fetal goitrous hypothyroidism that was successfully treated with intra-amniotic infusion of levothyroxine.

## 2. Case Presentation

### 2.1. Patient History

The patient is a 41-year-old female G5P3 (gravida 5, para 3) at 20 weeks gestation who was referred to maternal fetal medicine (MFM) by her obstetrician–gynecologist for advanced maternal age. She has a history of three prior uncomplicated vaginal deliveries and one miscarriage. She has no significant past medical history. Her only medication is aspirin 81 mg and prenatal vitamins taken daily. Her body mass index was 46.

### 2.2. Obstetrical Course

**Figure 1:**
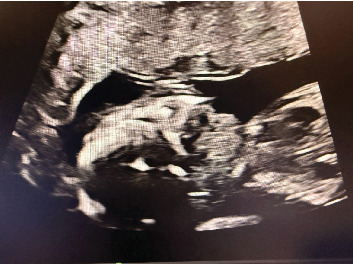
Imaging findings. 2D ultrasound of the fetus at 20 weeks gestation, showing an anterior fetal neck mass.

At her 20-week Level 2 ultrasound with MFM, the fetus was incidentally found to have an anterior homogeneous neck mass that was suspected to be fetal goiter (Figure 1). No other anomalies were seen. Thyroid studies were recommended for the patient, which showed normal levels of TSH, free T4, free T3, and thyroid stimulating immunoglobulin (TSI). The patient denies a history of thyroid abnormalities in herself and her three prior children. The patient was instructed to follow up to reassess the neck mass and rule out hydrops fetalis.

**Figure 2:**
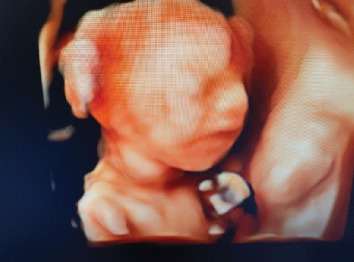
Imaging findings. 3D ultrasound of the fetus at 24 weeks gestation revealed anterior neck mass, measuring 3.3 × 2.1 × 1.4 cm.

**Figure 3:**
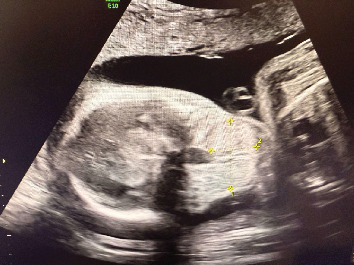
Imaging findings. 2D ultrasound of the fetus at 24 weeks gestation showing a fetal anterior neck mass.

Patient returned for her follow-up visit at 24 weeks for a repeat ultrasound, which showed the fetal neck mass to still be present and measuring 3.3 × 2.1 × 1.4 cm (Figures 2 and 3). Other findings included polyhydramnios and hyperextension of the fetal neck. The patient was counseled on the risks and benefits and offered options of cordocentesis, amniocentesis, or expectant management. She declined a cordocentesis to evaluate fetal TSH levels due to the potential risks of the procedure. She ultimately decided and consented to an amniocentesis with installation of levothyroxine. Her first amniocentesis for genetic screening was performed at 25 weeks gestation which showed normal results. The patient was recommended to have a fetal MRI for further evaluation.

At 26 weeks gestation, the patient underwent a fetal MRI which confirmed findings of fetal goiter with no other fetal anomalies. The mass measured 3.68 × 1.64 × 1.08 cm. The same week, the patient underwent amniocentesis with instilled infusion of 200 mcg of levothyroxine into the amniotic cavity. The patient subsequently returned weekly for amnioinfusion of levothyroxine. The dose of levothyroxine was increased to 400 mcg at 27 weeks gestation due to the goiter not shrinking in size. The assumed diagnosis at this point was congenital hypothyroidism causing goiter, or fetal goitrous hypothyroidism. Other differentials included (1) fetal hyperthyroidism, which was less likely due to the patient being euthyroid; (2) teratoma; (3) cystic hygroma/lymphangioma; (4) hemangioma; and (5) epignathus, which were ruled out with fetal MRI.

At each weekly visit from 26 weeks and beyond, the patient received amnioinfusion of levothyroxine into the amniotic cavity. Additionally, TSH and gram stains were collected during amniocentesis procedures. The patient was counseled that amniocentesis is not a perfect diagnostic tool for TSH as there are no standard reference ranges. The reference ranges are from case series of similar cases. Despite this, of note, the TSH in the amniotic fluid for this patient showed a downward trend, beginning at 0.71 IU/mL and stabilizing at around 0.2 IU/mL at the final amniocentesis.

**Figure 4:**
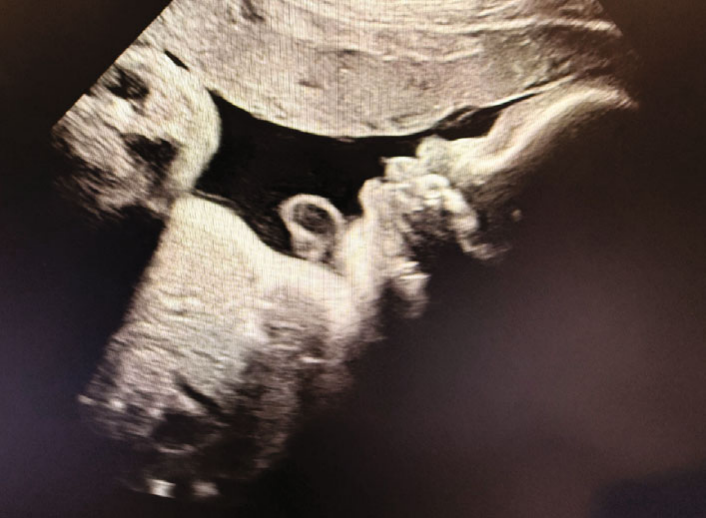
Image findings. 2D ultrasound of the fetus at 30 weeks gestation showing resolution of anterior neck mass and resolution of neck hyperextension.

The fetal goiter also shrunk with each subsequent visit and amnioinfusion of levothyroxine. At 28 weeks gestation, the goiter had started to shrink in size to 3.0 × 1.7 × 1.5 cm. During this week, the polyhydramnios had also resolved. At 29 weeks gestation, the goiter measured even smaller at 2.7 × 1.7 × 1.7 cm. By 30 weeks gestation, the goiter was no longer appreciated and the fetal neck was no longer hyperextended (Figure 4). The patient continued to receive 300 mcg of levothyroxine via amnioinfusion until 34 weeks gestation.

### 2.3. Delivery and Postnatal Course

Around 34 weeks gestation, the patient developed gestational hypertension and then preeclampsia. She had planned to have an induction of labor at 37 weeks, but her amniotic membrane ruptured spontaneously at 36 weeks, and she went into labor. She had an uncomplicated vaginal delivery and her blood pressure stabilized. There was no postpartum hemorrhage. The infant's APGAR scores were 9 and 9 at 1 and 5 min after birth. There was no goiter noted on the infant's physical exam. The infant followed up with pediatric endocrinology and was diagnosed with congenital hypothyroidism and started on levothyroxine. Both the infant and mother are doing well today, with the infant showing no signs of neurodevelopmental impairments.

## 3. Discussion

Although there have been a few similar case studies that showed reduction of fetal goiter with intra-amniotic infusions of levothyroxine in the setting of fetal goitrous hypothyroidism, there are currently no standard treatment guidelines for fetal goitrous hypothyroidism. Moreover, no standard dose of levothyroxine has been established, and the dose of levothyroxine in this case was tailored based on the response of the fetal goiter to treatment. In our case, intra-amniotic infusion with 200 mcg of levothyroxine did not show improvement in the fetal goiter size. However, 400 mcg of levothyroxine caused the goiter to reduce in size weekly, and subsequently resolve by 30 weeks.

Although cordocentesis is the current gold standard for diagnosing fetal hypothyroidism, our patient declined to undergo cordocentesis after risks and benefits were discussed with her. Therefore, we opted to measure TSH in the amniotic fluid, which is less invasive than cordocentesis. Although there is currently no standard reference range for TSH in the amniotic fluid [7], the TSH in the amniotic fluid of our patient did show a downward trend with intra-amniotic levothyroxine infusions. More studies are needed to determine the validity of TSH levels in amniotic fluid and establish a standard reference range.

In conclusion, this case demonstrates a successful treatment of fetal goitrous hypothyroidism with intra-amniotic infusion of levothyroxine. By treating fetal goitrous hypothyroidism with intra-amniotic infusion of levothyroxine, potential complications such as preterm delivery, the need for an ex utero intrapartum treatment (EXIT procedure), and possible neurodevelopmental impairments, as well as possible death of the neonate were avoided. More studies are needed to establish clear treatment guidelines and a standard dose of levothyroxine for amnioinfusion.

## Data Availability

Data sharing is not applicable to this article as no datasets were generated or analyzed during the current study.

## Patient Perspective

After the discovery of the fetal goiter, there was discussion of expectant management with the possibility of an increase in size and risks of polyhydramnios and preterm labor and the potential need for an exit procedure. Therefore, a recommendation to undergo amniocentesis and amnioinfusion was made, and the patient agreed although she initially was slightly anxious about the invasive procedure and associated risks, including potential pregnancy loss, infection, or preterm labor. These concerns were heightened by the emotional impact of learning about the presence of a fetal goiter. The patient was also counseled on the expected neonatal and obstetric benefits, including the possibility of avoiding respiratory compromise at birth and increasing the likelihood of an uncomplicated vaginal delivery, thereby reducing the need for a cesarean section or advanced neonatal airway interventions. She felt happy that she was able to be proactive and address the situation with treatment to help avoid some of these complications that could occur.

Upon understanding the potential for direct benefit to fetal health and delivery outcomes, the patient ultimately consented to the procedures. The interventions were well tolerated, and follow-up imaging demonstrated a decrease in the size of the fetal goiter. The pregnancy progressed without further complications, and the patient achieved a vaginal delivery of a healthy infant, without airway compromise or the need for surgical intervention. This case illustrates how shared decision-making, clear risk–benefit communication, and multidisciplinary support can lead to informed consent outlining the risks, benefits, and rationale for the procedures. Overall, she was very satisfied with the care and ability to treat her baby in utero.
